# Understanding Generation Gaps in LGBTQ+ Communities: Perspectives About Gay Neighborhoods Among Heteronormative and Homonormative Generational Cohorts

**DOI:** 10.1007/978-3-030-66073-4_14

**Published:** 2020-11-30

**Authors:** Alex Bitterman, Daniel Baldwin Hess

**Affiliations:** 17 Department of Architecture and Design, Alfred State University of New York, New York, USA; 18 Department of Urban and Regional Planning, University at Buffalo, Buffalo, NY USA; 19 Alfred State College, State University of New York, Alfred, NY USA; 20 University at Buffalo, State University of New York, Buffalo, NY USA

**Keywords:** Gay neighborhoods, Gay studies, Gayborhoods, Generations, Generational theory, Greatest Generation, Baby boomers, Generation X, Millennials, Generation Z, LGBTQ+

## Abstract

Using Strauss-Howe generational theory as a guiding structure, this chapter examines differences between generational identity for LGBTQ+ individuals compared to heteronormative generational identity. We theorize that LGBTQ+ individuals may identify with two generational cohorts—one defined by birth year and a second related to “coming of age” as a sexual minority. A case study examining the lifespan of four LGBTQ+ celebrity personalities demonstrates the concept of generational layering. We argue “generational layering” affects various aspects of LGBTQ+ life, including connection to place as reflected in attitudes of LGBTQ+ people regarding gay neighborhoods. The chapter concludes with five takeaway messages that clarify the relationship between LGTBQ+ people, the generational cohorts to which they belong and with which they identify, and the attitudes of various LGBTQ+ generational cohorts toward gay neighborhoods.

## Introduction

Generations give structure to society. Through engagement with our beliefs, behaviors, and values, we understand the world around us—and other people—based on our experience through a generational cohort with which we identify. As societies and cultures progress through time, generations are one metric by which humans organize shared experiences throughout history.

LGBTQ + people have been impacted by generational values and expectations and more recently have begun to engage generational identity differently than heterosexual peers. LGBTQ+ individuals do not always “fit” into the paradigm of their birth generation in the same way that heterosexual individuals do. As societies advance from one generation to the next, one measure of progress made toward equal civil rights can be seen in the changes in the attitudes and perceptions of LGBTQ + people. Typically, behaviors and values of each successive heteronormative generation reflect broadly-held opinions and behaviors of that generational birth cohort, including attitudes and views regarding LGBTQ + people and lifestyle. These prevailing opinions undoubtedly influence LGBTQ+ people. We argue in this chapter, however, that LGBTQ+  generations do not operate solely in concert with their “birth” generation. Instead, LGBTQ + individuals are dually influenced both by the heteronormative birth generation in which they are born and by the LGBTQ+ generation during which they “come of age,” which is related to “coming out” and forming a personal identity as an LGBTQ+  sexual minority . This “layering” or “dual-lens” through which people prescribe a generational label recognizes the multivariate attributes that shape generational behaviors and beliefs and overall worldview for LGBTQ + individuals.

In this chapter, we examine the generational saeculum of the past century and the relationship of each successive generation to the birth cohorts of the entire century. Just as the behaviors, attitudes, and values of each heteronormative generation are clearly defined, we argue that similar—but different— parallels can be claimed for LGBTQ + generational cohorts. Throughout, we develop a broad overview of birth generations and LGBTQ + generations as a model for how generational theory might be applied specifically for LGBTQ+ individuals and LGBTQ+ generational cohorts, in that the experience for LGBTQ+ is arguably different and shaped by “ coming of age” more so than for heterosexual people. Our aim is not to oversimplify or stereotype, but to construct a general guide to frame one potential perspective to better understand the homonormative experience in a heteronormative world. Through this refreshed understanding, we examine comparative cases that describe the biographies, general behaviors, and generational locus of four well-known gay men as a means to explore how individuals born in a particular birth generation may experience vastly different experiences in life due to the LGBTQ + generation with which they identify. This comparison provides a basis for better understanding broader societal forces that shape the evolution of gay neighborhoods throughout the twentieth century and into the twenty-first century along with observations about the perceived decline or plateau of gay neighborhoods.

## A Brief Overview of Generational Cohorts

A generation encompasses a cohort of people born over a defined two-decade span. Strauss and Howe (1991, 1998) describe a social generation as the aggregate of all people born over—approximately—a span of twenty years. Generations are identified (from first birth year to last) by grouping cohorts of this length that share specific criteria. Therefore, an individual’s birth generation is typically defined by the year of birth, and members of a birth generation share an “age location in history.” That is, members of the generation encounter key historical events and social trends occupying the same life phase. In this way, members of a generation are shaped in lasting ways by the significant world events they encounter as children and young adults. They share certain common beliefs and behaviors. Aware of the experiences and traits shared with their peers, members of a generation also share a sense of common perceived membership in that generation ( Strauss and Howe 1991).

**Fig. 14.1:**
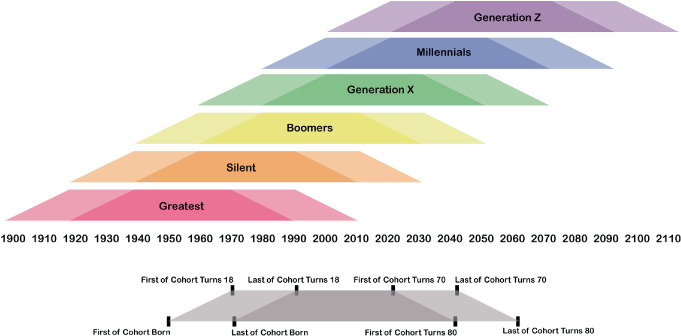
Generational cohorts between 1900–2100 (*Source* Graphic by authors)

**Fig. 14.2:**
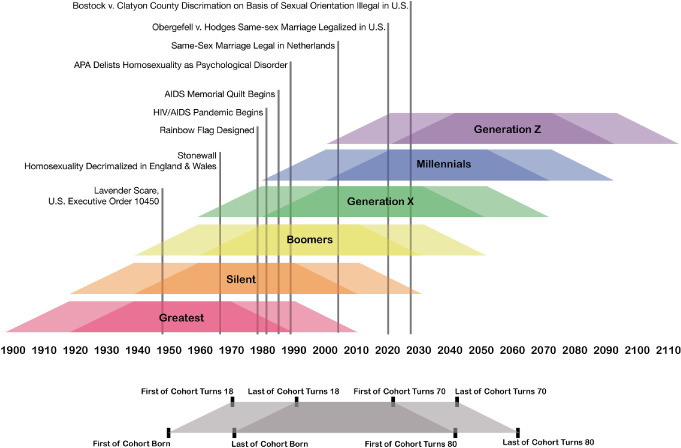
Generational cohorts and significant events for the LGBTQ + community (*Source* Graphic by authors)

 Generations are often influenced by formative events—war, famine, natural disaster, pandemic, economic upheaval, political unrest, etc.—that shape the behaviors of the individuals within that generation. Put another way; people become products of their time. For example, those born in the twenty years following the conclusion of World War II belong to the “Baby Boom” Generation and their lives were shaped by the end of the war, reconstruction efforts, and a shifting economic and geopolitical landscape. This generational worldview is a perspective through which life is framed over the lifespan. Just as people age independently, generations age in kind. Events throughout a generational lifecycle are signaled by benchmark years that correspond to the individual lifecycles of generational members. For example, the year the first of the cohort turns 18 years old, and the year the last of the cohort turns 18 years old, as shown in Fig. 14.1, signals the beginning of “adulthood” for that generation. This sliding scale of significant benchmarks frames the coming of age for a particular generation, which can intersect with significant world events that shape the values and impact the long-term outlook for that generation, as shown in Fig. 14.2. These events are important in that they influence not only human behaviors but also individual outlook and expectations throughout a lifespan.

A particular generation of people (born over a 20-year span) does not exist in isolation; each generation has interactions with the preceding and subsequent generations. Generations are organized in a series of four consecutive generations to comprise a “saeculum” which spans approximately 80 years, or roughly the duration of an average human lifespan, encompassing: childhood, young adulthood, midlife, and old age as shown in Fig. 14.1 ( Strauss and Howe 1991). Strauss and Howe (1998) note that broad generational patterns—archetypes among the saeculum—and historical events curiously appear to repeat in a relatively regular fashion over a lifespan and bear influence on the course of human history.

**Fig. 14.3:**
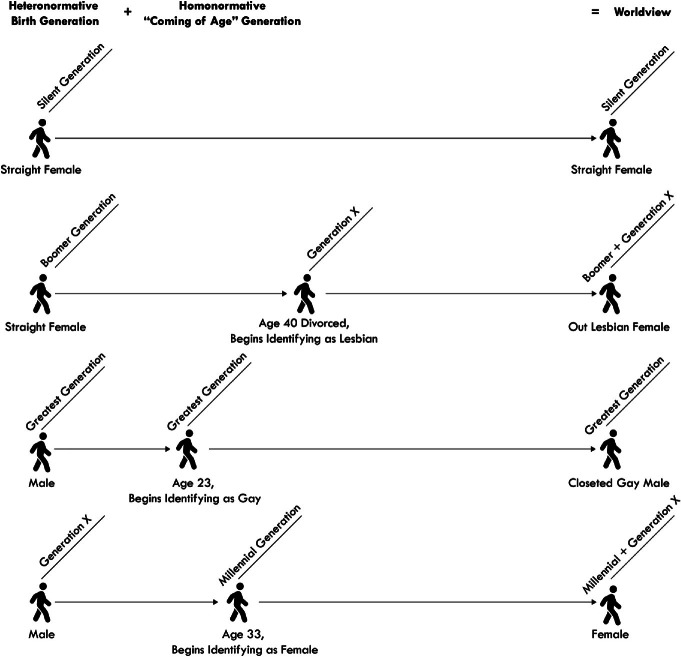
LGBTQ + individuals often identify with two generations: one defined by birth year and a second related to “ coming of age” as a sexual minority . The birth generation worldview is overlain by an additional LGBTQ + generational worldview (*Source* Graphic by authors)

Like all human beings, LGBTQ + individuals belong to a generational cohort according to their birth year. However, we argue that some LGBTQ+ individuals also identify with a *second* generational cohort, corresponding to the time of their coming of age. Whereas a birth year assignment to a generation assumes heteronormative behaviors across a person’s lifespan, coming of age (which can occur at any point over the lifespan) has sometimes greater importance than birth on how an LGBTQ+ individual expresses sexual orientation and identity, given the social influences and societal norms of that specific point in time. Therefore, LGBTQ + individuals belong to a birth generation and may also belong to a separate parallel LGBTQ+ generation based on the year the LGBTQ+ individual began to identify as a sexual minority . However, we argue LGBTQ + generations can also be delimited, distinct from broader heteronormative generational birth cohorts. As shown in Fig. 14.3, the homonormative experience is shaped as a summation of the values, experiences, and events that shape a birth generation plus the values, experiences, and events that impact that person relative to their coming of age as an LGBTQ+ individual. Because the coming of age or “coming out” moment may occur at any point along the continuum of the lifespan (as demonstrated by the Warhol, Hudson, Capote, Vidal case study below), the corresponding generational worldview for most LGBTQ + people is better defined by their coming of age than only by their birth.

## The Contemporary Heteronormative Saeculum and Events that Shaped the World

The analysis contained within this chapter encompasses six generations that span the end of one saeculum, the entirety of another saeculum, and the advent of a third. This period stretches across a four-century span from the very late 1890s to the 2100s. These six generations correspond with the time in which gay neighborhoods emerged, formed, matured, and plateaued (Hess and Bitterman 2021) and also encompass a future unknown at present.

 Heteronormative birth generations are relevant to gay neighborhoods but in a broader, more encompassing manner than homonormative LGBTQ + generations. General observations about the relationship of birth generations in relation to gay neighborhoods include:

- *The* *Greatest Generation* comprises individuals born between 1901 and about 1927, and many in this generation experienced World War I as children. Members of this generation experienced the Great Depression as early adults, and many participated in World War II. Freedom of gender expression or sexual orientation outside of the defined societal norm was highly unusual, and most LGBTQ + individuals were closeted during this period (Chauncey 1995).
- *The* *Silent Generation* includes those born between the late 1920s and the mid 1940s and is the last generation of the Great Power Saeculum (the span of generations from 1860 to 1945) ( Strauss and Howe 1998). Many in this generation experienced World War II or the immediate effects of the war as children. Little freedom or tolerance to express gender or sexual orientation outside of the defined societal norm defined this period (Chauncey 1995). However, the emergence of a secretive gay “code”—language, slang, and styles of dress—for identifying other LGBTQ + individuals began to emerge as a discernible subculture, especially in theatrical and circus professions (Baker 2020).
- *The Baby Boom* *Generation* comprises people born after World War II, from approximately 1945 to 1960, and is the first generation of the Millennial Saeculum, which spans from 1945 to the present. Many in this generation experienced the rise of the Atomic Age, the Cuban Missile Crisis, and the Vietnam War. They participated in the social revolution of the 1960s that gave rise to broader rights for women (Gencarelli 2014) and steadily increasing tolerance for LGBTQ + individuals, at least across Europe and North America. The sexual revolution and liberation of the 1960s loosened the social constraint on the expression of sexual orientation and gender identity, especially for LGBTQ+ individuals (Drasin et al. 2008). Though it was tolerated, homosexuality remained illegal in most jurisdictions through this period, and gay neighborhoods began to form in large cities as escapes from persecution and harassment (Lewis 2012).
- *Generation* *X* is composed of people born between the early 1960s and the early 1980s. Most in this generation experienced the Cold War, the birth of home computing, and the increasing digitalization of media. Some people in this generation were on the front lines of the HIV/ AIDS pandemic in the early 1980s, while others watched as the HIV/AIDS pandemic devastated the LGBTQ + population (Rosenfeld et al. 2012). During this time, LGBTQ+ characters began to appear on mainstream television, and laws prohibiting homosexuality in most Western societies were repealed or abolished. Gay neighborhoods became sites of organizing and activism for dignity and equality and against the systemic discrimination against LGBTQ + individuals in the wake of the HIV/ AIDS pandemic.
- *The Millennial* *Generation* includes those born between the mid 1980s and the early 2000s. Unlike previous generations, the social structure of the millennial generation focuses on flexibility, digital connection, and less association with institutions (Drake 2014). Millennials also witnessed as children the terror attacks of September 11, 2011, and throughout this period mass violence and terror attacks—including the Oklahoma City bombing in 1995, the Columbine High School shooting in 1999, the Paris terror attacks in 2015, the Tokyo subway sarin attack in 1995, and the London Westminster terror attack in 2018—became more prevalent and many among this consequently generation experienced anxiety and fears regarding personal safety (Alexander Agati 2012). “Helicopter Parenting,” a byproduct of the anxiety caused by a rise in perceived threats surrounding Millennials , increased the likelihood of overprotective parents and decreased the ability for children and young people to play outdoors unsupervised (Woolley and Griffin 2015). Millennials were also the first generation to begin to disregard notions of binary gender and destigmatize same-sex relationships (Jones et al. 2014); this was an essential step in increasing civil rights and protections for LGBTQ + individuals. The resultant plateau in gay neighborhoods may partly be attributed to the arrested development of this generation in which young adults live with parents longer (Tomaszczyk and Worth 2020; Bleemer et al. 2014) and an increased generational propensity to speak with parents about sexuality and sexual identity (Drumm et al. 2020).
- *Generation* *Z* includes individuals born from approximately 2005 to the present. Generation Z will be the last generation of the Millennial Saeculum. During this period, civil rights and legal protections for LGBTQ + individuals and same-sex marriage became increasingly prevalent (Jones et al. 2014) in Europe, Australia, North America, and parts of South America. However, homosexuality during this period remains illegal across much of the Middle East, Africa, and Asia, and civil rights and protections for LGBTQ+ individuals are few. Violence against LGBTQ+ individuals has reemerged in countries like Chechnya and Russia and renewed discrimination against LGBTQ+ people has resurfaced in countries like Poland. The relationship of gay neighborhoods to Generation Z remains unclear, as the oldest members of the generation are still too young to be living independently. Nevertheless, if trends with Millennials are an indication, then movement among younger people, in general, may begin to steadily decrease, which could impact the longer-term sustainability of gay neighborhoods.

## Exploring LGBTQ+ Generations: Through the Eyes of Warhol, Vidal, Capote & Hudson

Examining the lives of celebrities and well-known LGBTQ + individuals offers a lens to summarize and illustrate typical behaviors and attitudes that have been formative in shaping gay culture and the LGBTQ+ collective identity. Here we examine four well-known twentieth-century American personalities as a means to better understand the differences between LGBTQ+ individuals within the same generational cohort. By examining the events in the lives of LGBTQ+ individuals, we can better understand the formative factors that helped to support and shape gay neighborhoods.

**Fig. 14.4:**
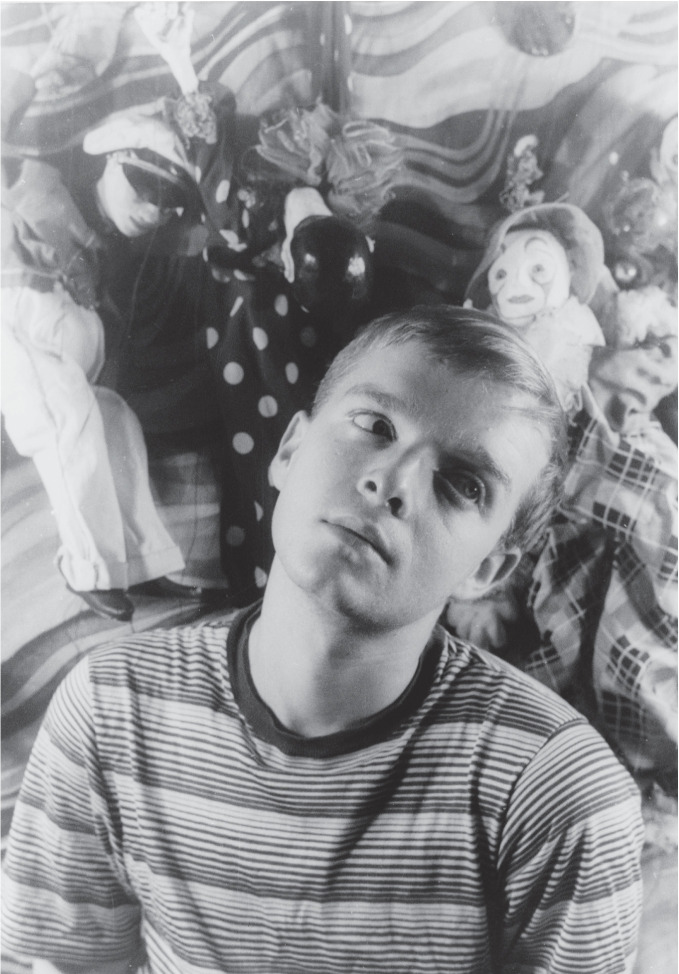
Truman Capote (*Source* Photo by Carl Van Vechten. Courtesy of: Van Cechten Collection, U.S. Library of Congress)

**Fig. 14.5:**
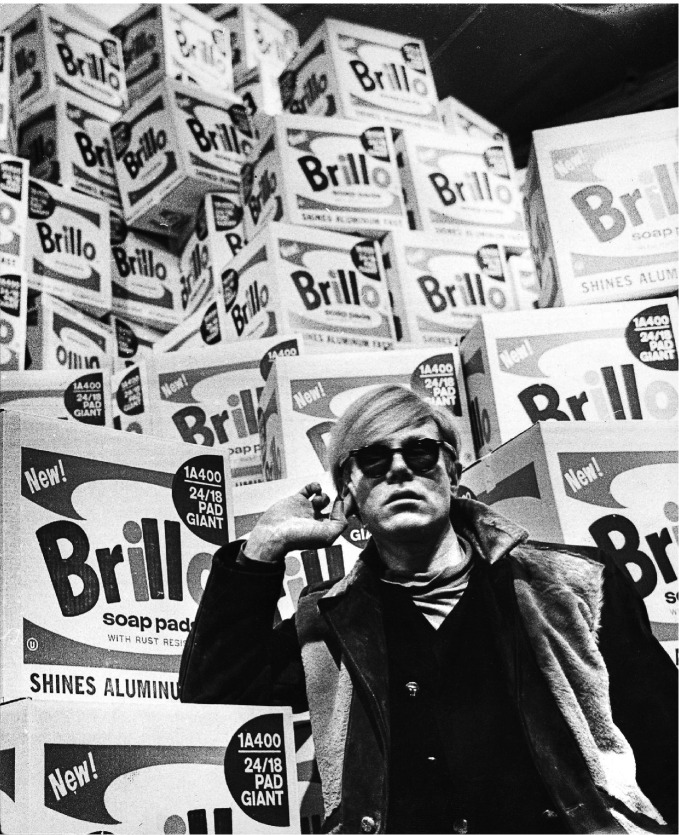
Andy Warhol in Moderna Museet, Stockholm, before the opening of his retrospective exhibition. Brillo boxes are seen in the background (*Source* Image courtesy of Lasse Olsson/Pressens bild)

**Fig. 14.6:**
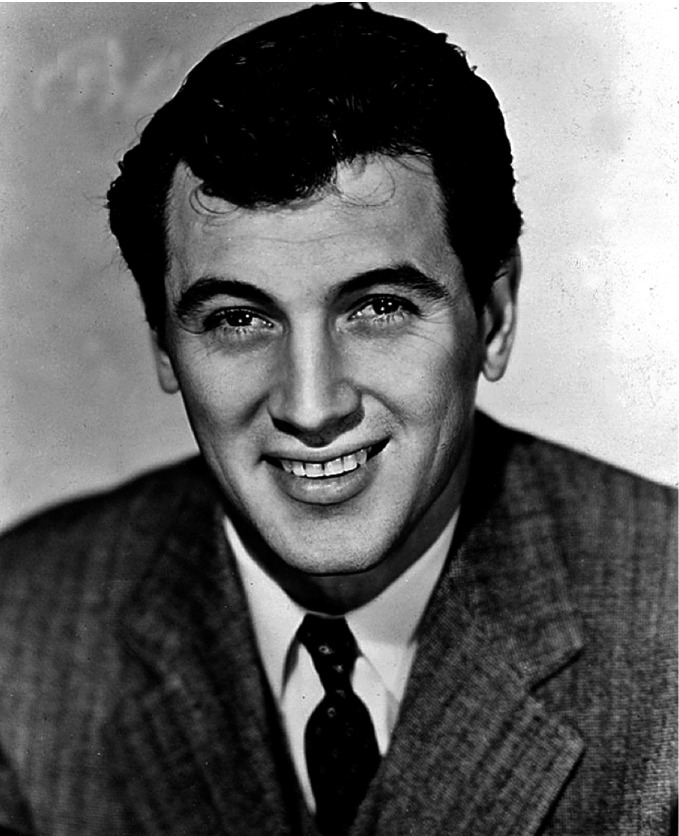
1952 Publicity photo of Rock Hudson from *Has Anybody Seen My Gal?* (*Source* Image courtesy of Universal Pictures)

**Fig. 14.7:**
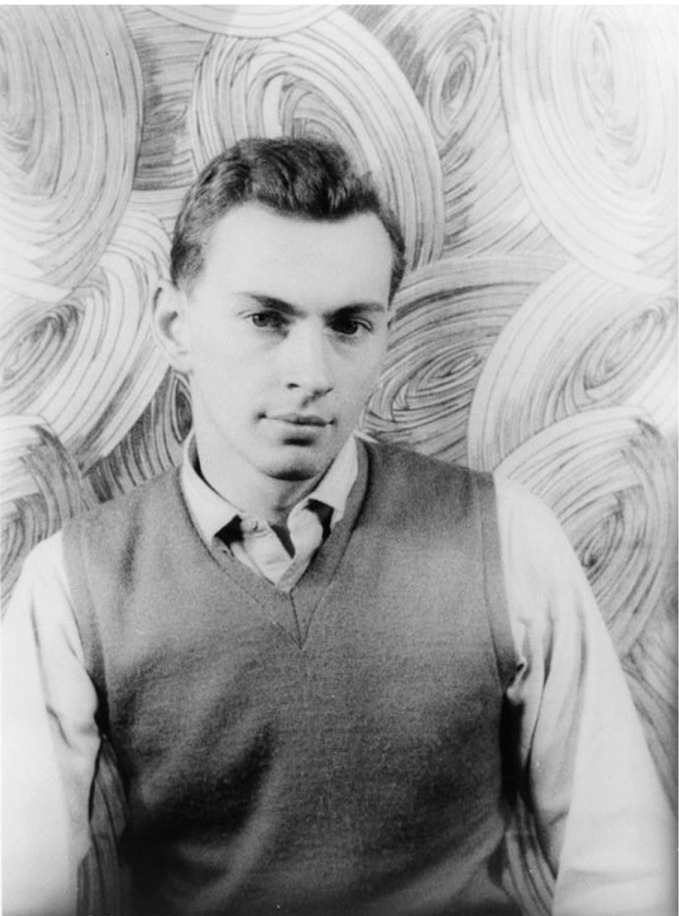
Gore Vidal (*Source* Photo by Carl Van Vechten. Image courtesy of: Van Cechten Collection, U.S. Library of Congress)

 Andy Warhol, Gore Vidal, Truman Capote, and Rock Hudson (see Figs. 14.4, 14.5, 14.6 and 14.7) were born during a four-year period, and all were members of the same birth generation. Despite the close proximity of their birth years, these men—and especially their LGBTQ + identities—were, in effect, generations apart. As noted, the social values and mores of a LGBTQ+ generation are not necessarily in alignment with the societal values and mores of a corresponding birth generation. In this case, the discontinuity between the birth generation to which each man belonged and the period during which their coming of age with regard to their LGBTQ+ identity occurred was shaped not only by the values, behaviors, and mores of their birth generation but also overlaid by the generation to which they “came of age” as a gay man and a member of the LGBTQ+  community. Exploring the lives of these four men helps illustrate the differences between LGBTQ + generational behaviors and the dissonance between what we term LGBTQ+ generational cohorts in contrast to birth generational cohorts (Figs. 14.4, 14.5, 14.6 and 14.7).

 Gore Vidal (1999, 2012) and Rock Hudson (Oppenheimer and Vitek 1987) were both born in 1925, and Truman Capote was born in 1924 (Long 2008; Dunphy 1987). All were members of the “ Greatest Generation ” of individuals born between 1901 and 1927. Each of the men is now known to have been gay. However, each came of age at different times, and they chose to publicly assert their homosexuality at a different time, influencing the manner by which they engaged their sexual orientation and expression. Capote was openly homosexual and had same-sex lovers from an early age (Long 2008). His dress and behavior—partly what underpinned his unique brand of celebrity (Long 2008; Dunphy 1987)—was less stereotypically masculine than either Hudson or Vidal. Capote was atypical of his heteronormative birth generation. His coming of age occurred early in life, which places his behavior, the outward expression of gender identity, and sexual orientation in a much more contemporary timeframe closer in behavior to a member of Generation X (people born about fifty years after Capote).

In contrast, Vidal did not publicly acknowledge his sexual orientation or gender expression, and much later in life vaguely identified first as bisexual (1999), and later as homosexual (Kaplan 2013). Though born of the same generation as Capote, Vidal’s behaviors were quiet (2012), his gender expression was comparatively cis, and he stayed consistent in behavior and presentation throughout his early life. However, he became slightly less guarded about his sexual orientation and more “out” as he grew older. Vidal was a typical member of his birth generation. Still, over time, his behaviors and attitudes became more distinctive and in line with an LGBTQ + member of the Baby Boom Generation —quiet and perhaps conflicted, but open to sharing his sexual orientation to those “in the know.”

For the better part of his life, Hudson did not publicly address his sexual orientation (Griffin 2020) but was a cis man and was straight acting in public. Moreover, Hudson actively denied rumors about his sexual orientation for much of his life (Oppenheimer and Vitek 1987), fearing being “outed.” He remained fully closeted until he became ill with HIV/ AIDS in 1984 (the same year Capote died). Hudson was one of the first major celebrities to be diagnosed with HIV/AIDS, and his coming out was implied de facto when he publicly revealed his HIV/ AIDS diagnosis in July 1985 (Oppenheimer and Vitek 1987). Hudson was born a member of the Greatest Generation and remained both a birth member and an LGBTQ + member of that generation for the entirety of his life.

Each of these three men, born within a year of one another, belonged to the same birth generation. Still, each chose to express his gender and sexual orientation differently, effectively coming of age with their LGBTQ+ identity at various points throughout their lifespan. Their behaviors, outward expression of gender, and degree of comfort with identifying as LGBTQ + varied depending more on their LGBTQ + generation than their birth generation.

As with Capote, Vidal, and Hudson, a desire or lack of desire to congregate and be associated with other LGBTQ + individuals in public impacted the emergence and subsequent development of gay neighborhoods. Initially, gay neighborhoods were populated by astereotypical individuals who did not “fit” into the predominant heteronormative society or were persecuted for their behaviors or beliefs. Capote is a prime example of such an LGBTQ+ individual, and throughout his lifetime, he was ahead of his time in being both publicly and privately “out.” Capote, arguably less cis than either Hudson or Vidal, frequented gay establishments and was regularly seen about town in gay neighborhoods in New York. Initially, during this time, gay neighborhoods were mostly the domain of “sissies,” “fairies,” or “ queers” (Gordon and Meyer 2007). Other LGBTQ + individuals avoided gay neighborhoods either out of contempt or fear of public association with LGBTQ+ people or the denigrative “ queer” label that was connected to those who frequented or lived in gay neighborhoods. Over time, however, gay neighborhoods diversified and became less homogeneous, and this diversity helped achieve freedom of association beyond the stereotype. Other LGBTQ+ individuals, perhaps less comfortable with being stereotyped as “fairies” or “sissies” (Fone 2000), began to participate in the vibrant LGBTQ + life the gay neighborhoods enshrined (Hanhardt 2013).

Another contemporary of Hudson, Vidal, and Capote—and a member of the greatest generation —is Andy Warhol. Born in 1928, Warhol defied all conventions, especially those related to gender identity and sexual orientation. Though he identified as homosexual, details regarding his relationships remain mostly unclear, even today (Gopnik and Halstead 2020). Throughout his career, Warhol was unique in that he completely disregarded any societal label for himself or others. Between the 1960s and the 1980s (throughout the latter part of his career), Warhol interacted socially and comfortably with a diverse spectrum of personalities (Gopnik and Halstead 2020; Koestenbaum 2015) including the überwealthy, celebrities, up-byand-coming stars, starving artists, and homeless Bohemians. Warhol also located his studio within or nearby various gay neighborhoods in Manhattan. In this way, Warhol’s liberal attitude mirrored attitudes in gay neighborhoods as home to not only LGBTQ+ individuals but as inclusive, accessible, and permissive neighborhoods where economic status became less important than creative energy, potential, and persona.

 Warhol, however, was a formative and formidable force in the shaping of gay neighborhoods, first as voyeur and then as provocateur and later as an observer and unintentional historian of sorts. Throughout his diaries, Warhol referred to evolving LGBTQ + urban spaces, especially in and around New York City, as gay neighborhoods began to become performative and public but safe places for LGBTQ+ people. In 1977, Warhol reflected on his daily life in New York City: “we walked around the Village. In the old days you could go over there on a Sunday and nobody would be around, but now it’s gay gay gay as far as the eye can see—dykes and leather bars with the names right out there in broad daylight—the Ramrod-type places” ( Warhol and Hackett 1989: 51). Later, Warhol reflected on his time in New Hope, Pennsylvania, noting that it was “90 percent gay. We went to a place called Ramona’s and a drag queen served us and people were drinking at 2:00 pm. Gay old guys. It was too gay for me, it drove me crazy. Like a time warp. A gay hotel-motel. The drag queen looked like Rupert’s mother with the blonde beehive. She had on pants but a four-inch leather belt really tightening in her waist…Then, we went to places run by gay sons and fat mothers. Antiques places” ( Warhol and Hackett 1989: 718). Warhol’s diary provides insight into the constellation of characters that participated in creating the gay neighborhoods of New York through the 60s, 70s, and 80s.

Despite his fascination with gay places and his high-profile interjection into gay neighborhoods, Warhol—despite his sexual identity—viewed himself as an outsider or observer (Koestenbaum 2015). “Gay” referred to other people, but in his mind, “gay” did not refer to him. The complexities of his self-identity, sexual orientation, and sexual expression were in ways well in advance of the time in which he lived. In this way, Warhol and his obsession with celebrity and cultural “influencers” and broad acceptance and documented fascination with others (Gopnik and Halstead 2020) defied his birth generation. His attitudes and behaviors are closer to Millennial behaviors than to his birth generation. However, regarding his own outward sexual identity, Warhol was very much typical of his birth generation—closer in behavior to Vidal and Hudson in viewing homosexuality as outside of his own experience, despite his engagement in same-sex relationships. The complexity of his coming of age in a time when homosexuality was illegal, mixed with his fascination with celebrity and outlandishness, sparked a curiosity in Warhol that helped to shape and support the culture of gay neighborhoods in New York City in the 1960s through the 1980s as inclusive and creative spaces. Through his art and signature publication *Interview* magazine, Warhol helped normalize same-sex relationships and LGBTQ + culture and construct a public face and voice for his followers—subsequent generations of LGBTQ+ individuals. He provided for his followers and for successive generations of LGBTQ+ people a type of freedom that he himself seemed reluctant to engage.

## The Homonormative Saeculum and the Events that Shaped a Century of LGBTQ+ Culture

The experience for LGBTQ + people—framed by the understanding and treatment of LGBTQ+ individuals reflected in the values of mainstream society—is often quite different from that of non LGBTQ+ people. Various degrees of implicit or explicit discrimination have existed (and continue to exist) for LGBTQ+ people. Attempts by LGBTQ+ individuals to “fit in” to—or find safe space among— heteronormative society vary based on birth generation and other factors. In heteronormative society, an individual is influenced by the events of the world, but in homonormative society, the formula is compound. Individuals are shaped by the events of the world, layered by fear or apprehension about how LGBTQ + people are treated (or mistreated) by society at large and the perception (or observation) of how LGBTQ+ people are received by an individual’s immediate social circle. Therefore, clarifying the experience of a “gay generation” could also shed light on the attitudes and behaviors of LGBTQ + individuals and even the degree to which LGBTQ+ engage gay neighborhoods and gay space. We propose appending the heteronormative generational names popularized by Strauss and Howe to better incorporate LGBTQ+ experiences as follows:

- The *Silent Generation* —*or the “Closeted Generation”*—gay men came of age just before, during, and immediately after World War II and lived in a world in which there was intense social pressure to conform to gender stereotypes. For many gay men, the choice to outwardly identify as gay was not an option, and doing so meant risking stigmatization, harassment or shunning (Bergling 2004). For this generation, gay—for men—equated with feminine characteristics—suggestive of the “lesser” sex—and the pejorative taunts “ fairy” and “ sissy” were used to denigrate the masculinity of gay men. Homosexual relations for this generation were illegal, and being discovered or “outed” as a homosexual could bluntly end a career and ruin social standing. The social stigma against gay men was strong, and few gay and queer men willingly chose to endure pressure or harassment.
  Consequently, few gay men chose to be “out” during this era. Those that did often fled to larger cities like New York and San Francisco. To avoid persecution and harassment by the police, these early pioneers further gravitated within these large metropolitan areas to the margins of central cities—abandoned and forgotten neighborhoods populated by those that heteronormative society has labeled social outcasts and criminals—that became some of the first recognizable gay neighborhoods. These neighborhoods were diverse, inclusive, and tolerant. Residents of these early gay neighborhoods banded together to protect each other and fight against a sometimes oppressive social culture.
- *LGBTQ * + *individuals born during the Baby Boom* *Generation* —*the “Liberation Generation*.” Gay men from this generation matured during the 1960s and 1970s. Many more outwardly expressed their sexual orientation (compared to the previous generation), though being clandestinely gay but still “in the closet” was common (Morrow 2001). High profile gay men hid their sexual orientation for fear of being “outed.” Remnants of the social stigma and shame prevalent during the previous generation persisted. However, the social turmoil of the late 1960s led to a broad social and sexual revolution in the 1970s (Troiden and Goode 1980).
  *The* *Homosexuals*, was a 1967 documentary produced and aired by CBS and hosted by Mike Wallace who framed homosexuality as an illness. Wallace interviewed guests who supported this claim and further edited the interviews to reinforced his supposition that homosexuality was a deviant illness. One retrospective review of the program noted *The Homosexuals* was “the single most destructive hour of antigay propaganda” in American history (Besen 2003: 227). The show “not only had a devastating effect on public opinion but also was a nuclear bomb dropped on the psyches of gay and lesbian Americans, who, prior to this show, had never been represented as a group on national television” (Besen 2003: 201).
  However, by the late 1970s, gay men began to appear in popular mainstream culture. On television, Lance Loud in *The Loud Family* and Billy Crystal in *Soap* helped to introduce mainstream audiences to gay characters, not as Disney villains, deviant criminals, or effeminate stereotypes, but as “normal” individuals. Despite vibrant private lives, many high-profile gay men, such as Andy Warhol, lived during this time “quietly” (i.e., publicly “in the closet”). Soon the “gay liberation” movement began. These contemporaneous social movements were considered progressive and permissive. Free love, equal rights, and expanded civil rights helped to buoy rights for LGBTQ + individuals. Despite the tumultuous transition, the winds of change had begun to blow for the LGBTQ+  community during this period (Duberman 2019).
- *Generation* *X* —*the “Out” Generation*. The experience of Generation X was markedly different than previous generations with regard to homosexuality. By the 1980s, mainstream acceptance of homosexuality was beginning to grow—slowly—but social pressure against homosexuality remained. Gay slurs became part of typical teenage slang, but some members of this generation braved societal disdain and disapproval and chose to live publicly as gay men or lesbian women. They were bolstered by the experiences of those from previous generations as they began to shed the cultural shame that encouraged LGBTQ + individuals to stay in the closet, and they relished in the outcomes of the gay liberation movement as gay and lesbian individuals and their allies began to celebrate “gay freedom.” During this time, LGBTQ + individuals tentatively began to find a collective voice, however mainstream heteronormative attitudes prevailed. Systemically and in comparison to today, bullying was more common and more tolerated; the notion of learning to “stand up for yourself” in the face of adversity was prevalent, and gender stereotyping was only starting to be examined.

Additionally, LGBTQ + members of Generation X were thunderstruck by the emergence of the HIV/ AIDS pandemic. Acceptance of LGBTQ+ individuals was framed in part by a sympathetic mainstream public disappointed and outraged by a lack of government acknowledgment and response and a blithe refusal to confront suffering brought about in the early days of the AIDS pandemic. Rock Hudson, a high-profile Hollywood heartthrob famous in the 1950s and 1960s, publicly revealed his HIV positive status and complications from AIDS (Griffin 2020; Oppenheimer and Vitek 1987). This news was met with icy silence by his longtime friends, then-President Ronald Reagan and First Lady Nancy Reagan. As “ safe sex” became a topic introduced to most high schoolers in health education courses, so too was—for the first time in any sanctioned capacity—the implication of homosexuality. High-profile efforts such as AIDS Coalition to Unleash Power ( ACT UP), Broadway Cares/Equity Fights AIDS, and the AIDS Memorial Quilt Project helped to forge public awareness of the societal and institutional marginalization of homosexuality and the necessity to address the AIDS pandemic with facts and not with fear. At the same time, other organizations fought to denigrate LGBTQ + individuals and against funding to find a cure for AIDS.

Generation X took notice of members of the Greatest Generation and Silent Generation as they struggled—often publicly—to reconcile the conflicting values of their generations: to acknowledge homosexuals as productive members of society while admitting that previous treatment of LGBTQ + people may have been unkind or immoral.

In contrast to previous times when popular cultural references implied shame or deviance related to homosexuality, many of the cultural touchpoints for Generation X viewed homosexuality as a “normal” part of society, suggesting an opening for the acceptance of LGBTQ + people. During the formative years of development for Generation X, psychologists and mental health professionals debated clinically normalizing homosexuality. As recently as 1968, the APA listed homosexuality as a mental disorder. In 1973, the American Psychiatric Association (APA) asked all members attending its convention to vote on whether they believed homosexuality to be a mental disorder: 5,854 psychiatrists voted to remove homosexuality from the list of mental disorders, and 3,810 voted to retain it. The APA compromised, removing homosexuality from the list but replacing it with the label “sexual orientation disturbance” for people “in conflict with” their sexual orientation. In 1987, the APA removed homosexuality as a classified mental disorder (Burton 2015; Mayes and Horowitz 2005; McCommon 2006; Rissmiller and Rissmiller 2006).

Simultaneously, the evolution and quasi-normalization of homosexuality played out for Generation X in popular culture. Pedro Zamora, who was both gay and HIV+, became one of the first openly gay reality television stars. He appeared on *The Real World*, then a wildly popular show and generational touchpoint which aired on MTV. Zamora introduced Generation X to being gay, out, *and* proud of it. Shortly after, Ellen DeGeneres made television and social history in 1997 when both she and the character she played in her eponymous television show came out as a lesbian. Changes in societal norms, reflected in popular culture, aided mainstream and heteronormative audiences to better understand LGBTQ + individuals as compassionate human beings and not as stereotyped gay caricatures. By the early 2000s, LGBTQ+ culture had begun to fuse into mainstream culture—still relegated to an unequal place, but proudly present at the table (Johnston 2017). During this generational period, gay liberation had advanced to gay freedom and eventually became gay *pride*.

- *The Millennial* *Generation*—*the “Proud Generation”* are those born between the mid-1980s and the early 2000s and followed Generation X . LGBTQ + individuals in this cohort and came of age at the beginning of the new millennium were less concerned with previously entrenched stigmas and stereotypes (MetLife Mature Market Institute 2010). Members of the Millennial Generation were more likely to be “out and proud” and socially more accepted than previous generations. Homosexuality became increasingly more accepted by heteronormative society during the period as this generation came of age, culminating in the legalization of same-sex marriage in Canada in 2005, Sweden in 2009, and the United Kingdom in 2013; in the United States, legalization of same-sex marriage first occurred state by state, but eventually the U. S. Supreme Court decision in *Obergefell v.* *Hodges* (2015) legalized same-sex marriage nationwide (Hart-Brinson 2018). However, in sharp contrast to members of the Silent Generation , Greatest Generation , Baby Boom Generation , and Generation X , LGBTQ + Millennials have had far fewer societal roadblocks to express their gender orientation and sexual orientation and are more likely than members of previous generations to describe fluidity or changes in sexual orientation over time (Vaccaro 2009). Further, they have come of age during a time of political correctness and comparatively low tolerance of behaviors that fuel stigma and division—bullying, racism, and sexism. Because of the lesser exposure to social friction for LGBTQ + members of the Millennial Generation, may LGTBQ+ gays and lesbians are understood by members of other generations to be blithely unaware of the persecution, harassment, and struggles endured by predecessor LGBTQ + individuals. In this way, LGBTQ+  Millennials are seen by others to take for granted their equalities and freedoms, which were fought for by LGBTQ + people who came before them.
- *Generation* *Z* —*the “Fluent Generation”*— The newest generation, Generation Z, completes the present saeculum and includes those born between 2005 through today. The behaviors, values, and perspectives of Generation Z are different from those of preceding generations (Archer 2012), shaped in part by the connectivity provided by digital technologies (Mowlabocus 2016) and the ability to form and participate in virtual communities using social media (MetLife Mature Market Institute 2010). Generation Z came of age in a period of expanding rights for LGBTQ + individuals punctuated by landmark legal cases such as the U.S. Supreme Court case *Bostock v.* *Clayton County*, in which the Court held that Title VII of the Civil Rights Act of 1964 protects employees against discrimination (Jurva 2020) on the basis of sexual orientation or gender identity. Today, these young people are likely to find a more open space for discussing their sexual orientation with family, parents, and mentors at a young age (Dean 2014). Furthermore, they will find greater acceptance as they explore various paths related to sexual orientation and sexual identity. They are unlikely to be subjected to the same degree of heteronormative social stigma of generations past related to status as a sexual minority person.

## The Intersection of LGBTQ + Generational Cohorts and Gay Neighborhoods

Why is *place* so important for young gay people? During a “coming out” or “ coming of age” related to sexual identity, many people leave an oppressive place in which they find themselves which may include separating from family, siblings, or parents. Many people explore their sexual identity as teenagers or college-age students and then move to a new place to begin their adult life. Place, in this way, becomes vital in self-selecting community and expressing personal values along with sexual identity. For LGBTQ + people, this transition may be especially important as young people transition from parental and familial control to making their own decisions in adulthood, which underscores the layering for LGBTQ + individuals of birth generation and “ coming of age” generation.

**Fig. 14.8:**
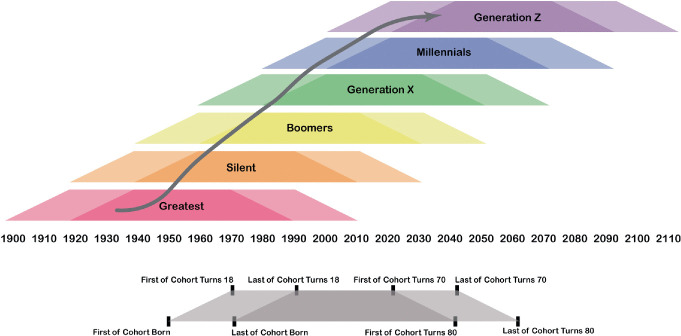
LGBTQ + generations and neighborhood change (*Source*: Graphic by authors)

The energy young adults bring to gay neighborhoods is the consistent (Bitterman 2020a). This energy is also the constituent that frames LGBTQ + generations, which helped to shape the gay neighborhoods in existence today. The desire among LGBTQ + individuals to live in a community such as those found within gay neighborhoods has been consistently evolving and changing over the past five generations, and the influx of young adults from each LGBTQ + generation, along with their energy and ideas helps to sustain gay neighborhoods for the next generation, as shown in Fig. 14.8.

While the popularity of specific neighborhoods may wax and wane with generational attitudes and values, the overall trajectory has been an upward one. In Fig. 14.8, the present moment is depicted as a plateau. The stewardship and forward momentum of gay neighborhoods has consistently been in the care of members of the previous generation who have “come of age” and then handed down to younger members of subsequent generations. This graphic suggests that gay neighborhoods began to emerge following World War II, fueled by the Greatest Generation members as they returned from fighting World War II (Chauncey 1995). Substantial growth continued through the 1950s and 1960s as members of the Silent LGBTQ + generation came of age and again through the 1970s as members of the Boomer Generation came of age. The period from the 1960s to the 1980s is often referred to as “the great gay migration,” when many LGBTQ + individuals moved to cities to establish their lives. The baby boomers fueled a period of sharp growth in gay neighborhoods during the 1980s and 1990s before LGBTQ+ members of Generation X had come of age. Growth continued until about 2000 as LGBTQ + members of Generation X came of age, but has plateaued since LGBTQ+ members of the Millennial generation have started to come of age.

Birth generation attitudes persist throughout a person’s lifespan, and values—the embodiment of these attitudes—are typically formed early in adult development. For LGBTQ + individuals, these values may shift or be overlaid by values of the LGBTQ+ generation to which they later belong. While societal mores change over time, generations provide constant frames of reference, and a “worldview” that remains tethered to a generational cohort. The case study of Hudson, Capote, Warhol, and Vidal illustrates the disassociation between birth generation and LGBTQ + generations. The difference for most LGBTQ+ people is that the product is typically more complex and multifaceted as the generational touchpoint is rooted in a heteronormative society.

The complexity of gay identity during the middle to later twentieth century—borne of generations influenced by social values and cultural mores instilled in their parents by their parents a century before—resulted in a conflicted state of existence for gay neighborhoods during their emergent and formative years. Those who frequented, inhabited, and visited gay neighborhoods balanced a personal disassociation with their LGBTQ + status, persistent cultural judgment and shame, and a desire for discretion with the freedom to express their true feelings through cautious participation and permissiveness. Older generations of LGBTQ+ pioneers helped build gay neighborhoods as safe spaces unthreatened by the harassment and persecution of a hostile world (Bitterman and Hess 2021). These respites provided fertile ground for a first generation of pioneers to organize, mobilize, and activate a wave of advocacy for LGBTQ + recognition and rights. These trailblazing generations shifted the public perception of “being gay” away from illegality and dereliction toward tolerance and normalcy. The societal stigma attached to being gay was magnified during the HIV/ AIDS pandemic—and the adversity experienced by gay men during (and after) that pandemic—shaped a generation of LGBTQ + activists, pioneers, and allies (Bitterman and Hess 2021). Challenging those in power and the institutions of power was no small effort for these trailblazers. Gay neighborhoods served as the geographic centers of a cross-generational movement, and gay neighborhoods remain essential to the shared cultural memory of the struggle for dignity, rights, and civil protections for LGBTQ + individuals. These hard-won aspects underpin LGBTQ + pride celebrations today. The uneasy balance of identity and gay neighborhoods common among the Greatest Generation was quickly torn apart by Baby Boom leaders in gay neighborhoods during the HIV/AIDS crisis. The stigma and pretense quickly evaporated to ensure survival. However, as later generations came to more broadly tolerate LGBTQ + individuals, the judgment and stigma of LGBTQ+ individuals did not immediately dissipate. Gay neighborhoods during this period from 1980 to 2000 provided a respite for LGBTQ + people—and especially gay men—from heteronormative standards and judgment based on the associated expectations.

Gay men from three generational cohorts—the Silent Generation , the Greatest Generation ( like Warhol, Vidal, Hudson, and Capote) and Generation X —were part of the “great gay migration” to cities in the 1960s through the 1980s (Weston 1995). People from marginalized groups could feel more comfortable, more accepted, and freer in large urban centers. After they migrated to large urban centers, they found themselves settling in gayborhoods: businesses—especially bars, restaurants, and cafes—catered to this captive audience. While most gay neighborhoods have historically been welcoming and inclusive to nearly everyone, the majority of gay neighborhoods were predominantly home to gay men. At the same time, the bars, cafes, and businesses supported a broader constituency under the LGBTQ + umbrella (and, later, non-LGBTQ + people). Lesbian women and other LGBTQ+ individuals tended to live elsewhere, and some viewed gay neighborhoods as gay “male” space. For example, bars and nightlife provide one example of the differences in inclusive and exclusive LGBTQ+ space common in the near past.

Until about 20 years ago, most LGBTQ-friendly bars tended to cater to one shade of people beneath the LGBTQ + umbrella. The target market became part of the identity of the bar (“lipstick” lesbian women, “ twink” [i.e., young] gay men, “bears,” etc.). While welcoming, in general, lesbian bars were not frequented by gay men; lesbian women also did not typically frequent gay bars, and so on. However, gay bars became increasingly “ gay-friendly” by actively welcoming allies and friends of the LGTBQ+  community. In this way, the bars became less exclusive and more inclusive (and today most welcome everyone—including those who do not identify as LGBTQ+) but are notably “ less gay.” This specific division common among bars in gay neighborhoods originally meant that the many stripes of the LGBTQ + community had individual space within a larger shared domain: the gay neighborhood. Similar observations could be made about cafes, restaurants, and shops in gay neighborhoods.

A loss of regular neighborhood bars has reduced social mixing opportunities among LGBTQ+ people from various generations (Bitterman and Hess 2021; Eeckhout et al. 2021). While previous generations of gay men preferred to socialize in bars visited strictly by gay men, those attending parties in gay neighborhoods today seek inclusive “ gay-friendly” dances and events (Eeckhout et al. 2021): “the relatively exclusive, niche-specific, semi-public spaces of lesbian and gay bars that promised a safe haven in a largely hostile environment lost their raison d’être faster than anyone would have expected a few decades ago” (Eeckhout et al. 2021, 238). These changes in how LGBTQ + individuals socialize in gay neighborhoods underscores broader societal shifts among younger generations (Bitterman and Hess 2021).

Between 2000 and 2020, some gay neighborhoods have appeared to plateau in popularity and use. The reasons for this perceived plateau are many and explored elsewhere throughout this book (Hess and Bitterman 2021). One notable shift is younger members of the Millennial and Z generations (who participated less directly in the struggle for LGBTQ + rights) may not fully grasp the importance of gay neighborhoods on LGBTQ+ culture and lesbian and gay life (Bitterman and Hess 2021) and may have a lesser propensity to engage in the community offered by gay neighborhoods. This may signal an emerging shift or potential disconnect between older and younger LGBTQ + generations, especially as fluidity in gender expression and sexual orientation shifts LGBTQ+ identity among the younger generations (Bitterman and Hess 2021). Effectively, for younger generations, making mainstream and heteronormative neighborhoods “ more gay” is more desirable than simply gravitating to existing gay neighborhoods. The result is that gay neighborhoods, as members of later generations, begin to pull away and become “ less gay.”

With these shifts, some anxiety has arisen among the denizens of LGBTQ + neighborhoods about the perceived demise of the incidental physical importance of these spaces, which may have interrupted the continuity among LGBTQ+ generational cohorts and accentuated the disconnects between various groups under the LGBTQ + umbrella (Bitterman and Hess 2021). The closure of gay bars, emerging virtual gay spaces, and changes in the character of gay neighborhoods are reminders that as these places transition from being home to generations rooted in the struggle, to playgrounds of generations benefitting from that struggle, now may be a critical time to examine the present plateau in the trajectory of gay neighborhoods (Coffin 2021). These younger individuals may view gayborhoods as relics of the past or may find gay neighborhoods not to be welcoming in ways that match contemporary sensitivities toward inclusivity (Bitterman and Hess 2021).

 Gay neighborhoods provide one means for examining generational evolution and change, and perhaps most acutely reflect a discontinuity between value and the need/desire for shared place. Gay neighborhoods also provide a physical location for capturing LGBTQ + cultural history and provide community support for organizations that capture and commemorate this history. Memory is short from generation to generation in relaying shared experience and collective history. Despite claiming to be motivated by the struggles of past generations (Hall-Kennedy 2020), members of more recent LGBTQ + generations often are unaware of specific details of the struggles and challenges encountered by previous generations, partly because these (typically) oral history details remain largely unrecorded and the places associated with the historical record are usually not fully documented or commemorated (Miller and Bitterman 2021). Unrecorded, the resultant collective wisdom forged by banding together as a community to overcome shared challenges risks being lost as moments pass into history. Over time, this transition away from an instigating problem may cause younger LGBTQ + individuals to take for granted the freedoms, acceptance, and rights hard-won by previous generations of LGBTQ + people (Bitterman and Hess 2021). This discontinuity can shift behaviors and the focus of immediate importance from one generation to the next and contribute to a loss of community and perception of relevance for gay neighborhoods.

A lack of continuity and awareness may threaten the existence (Podmore 2021) and the lasting value of gay neighborhoods (Miller and Bitterman 2021). In the United States, a national effort was started during the Obama administration to identify, memorialize, and landmark sites that provide significance to the history of the LGBTQ + community (Miller and Bitterman 2021). This important endeavor was intended to affirm the critical importance and relevance of these sites for generations to come (Bitterman and Hess 2021). The survival of smaller gay districts (and gay districts located in small- and mid-sized cities) is more threatened than established gay districts in larger metropolitan areas (Ghaziani 2021), and some locations have informally commemorated LGBTQ + significant places within or near gay neighborhoods.

## Future Possibilities for Gay Neighborhoods

The perspectives regarding gayborhoods among successive generations of LGBTQ + residents is changing. Attitudinal perspectives among generations are one significant factor in shifting demand for gayborhoods among LGTBQ+ groups. We believe that the inter-relation of these factors both shapes and reshapes the lived experience for LGBTQ+ people in neighborhoods and cities. As the stigma associated with identification with groups under the LGBTQ + umbrella decreases universally, the need/desire for living in places underscored by segregation and self-isolation may also change.

The physical building blocks of gay neighborhoods—commercial establishments ( bars, restaurants, bookstores), services ( community centers, health clinics), and residences—may be removed or displaced due to various urban forces including neighborhood change, revitalization, gentrification, socio-cultural influences (tastes, preferences, and attitudes), and even equal rights legislation (Bitterman 2020a; Eeckhout et al. 2021; Hess 2019, Hess and Bitterman 2021). However, if gayborhoods (or elements of gayborhoods) are at risk of or indeed disappearing, then the need to preserve these memory spaces becomes urgent so that the social action that occurred there is documented, (Miller and Bitterman 2021) especially for future generations.

Today, many LGBTQ + individuals—especially younger groups of individuals—embrace a broadly inclusive definition of sexual orientation and find little value in labels such as “gay,” “ lesbian,” “transgender,” and other sexual minorities (Podmore 2021). These younger individuals may view gayborhoods as relics of the past or may find gay neighborhoods not to be welcoming in ways that match contemporary sensitivities toward inclusivity (Bitterman and Hess 2021). Similarly, the older residents in gayborhoods are often less comfortable with the sexual diversity that younger people easily accept or the sexual fluidity they may practice. It can be difficult to distinguish between queer and hipster (Podmore 2021), and the hipster aesthetic marks gayborhoods as distinctly non-heteronormative space. For non-LGBTQ + individuals, “the idea that sharing space with hipsters serves to disrupt heterosexual norms and to recode the spaces as progressive, creative and open” (Podmore 2021, 304) underscores the generational shift with regard to gay neighborhoods. This is not a new phenomenon, as illustrated by the example of how Andy Warhol engaged the gay neighborhoods of New York and the various types of individuals that found a sense of belonging there.

Sexual fluidity among later generations shifts the generational perspective of gay neighborhoods (Bitterman and Hess 2021). Among those traditionally not found beneath the LGBTQ + umbrella, gender fluidity and diversity of gender expression—long conflated with “being gay”—has become more clearly articulated and is becoming more socially accepted. Shifting perceptions of gender, gender identity and fluidity, and gender expression—paralleling the rise of “ gay-friendly” culture—have given a broader mainstream voice to queer culture (Seidman 1994). We now live in a post-binary multi-polar world, and this change is reflected in neighborhoods and places (Hess 2019).

One example of the shifting language surrounding LGBTQ + identity is the familiar amalgamation of words that reference homosexuality as a cultural touchpoint, which are becoming increasingly common. For example, “ metrosexual”—a straight male with grooming or fashion-conscious characteristics typically associated with gay men—is one example of this cross-over. Similarly, a “ lumbersexual” is a homosexual with specific “butch” characteristics (manner or dress) reminiscent of a lumberjack. “ Cuomosexuals” are those individuals who appreciate the efforts of New York State Governor Andrew Cuomo, especially in fighting the COVID-19 pandemic (Miles et al. 2021). In contrast to the “ de-gaying” of gay neighborhoods, this shift could be considered the “gaying” of heteronormative society.

The increased precision of language to describe LGBTQ + individuals represents significant changes in worldview and perspective led by later generations who embrace less prescriptive and less rigid descriptors related to gender and sexual orientation. Observing the more recent blurring of differentiation between queer culture and hipster culture in the gay village of Montréal. Podmore (2021, 303) argues that “the boundaries between hipsters and queers were blurred rendering all young people in Mile-End as queer.” As generational thinking related to the expression of identity changes, this will likely alter gay neighborhoods and, indeed, all neighborhoods (Bitterman and Hess 2021), though the long-term effects of these changes remain unclear.

Perhaps “second generation” gay neighborhoods will serve future cohorts of LGBTQ + residents, citizens, families, and visitors by providing similar (and perhaps new, unimagined) functions just as established gay neighborhoods have served past generations (Bitterman and Hess 2021). While not all “seed” communities will flourish and external forces may even extinguish some, it is likely that as the needs of LGBTQ + citizens and families change, so too do the types of neighborhoods these citizens and families require as gay neighborhoods potentially reconfigure for the future. In this way, gay neighborhoods could reconstitute around the archetype, reflecting their existence for the previous five decades or in a form that does not yet exist. Moreover, we anticipate that established gay neighborhoods will propagate via an “ afterglow” (Coffin 2021) as historically relevant sites become landmarked or memorialized (Miller and Bitterman 2021). We expect to see new types of gay communities emerge in the future, especially as the Baby Boom Generation and Generation X (and subsequent generations) age into retirement (Hess 2019; Bitterman and Hess 2021). However, these neighborhoods may be different than those we know today.

## Synthesis and Conclusion: Connections for LGBTQ + People Across Generational Cohorts

To conclude this chapter, we synthesize the material presented to develop five takeaway messages. The takeaway messages underscore a layered approach to interrogating generational theory related to LGBTQ + individuals and experiences in gay neighborhoods. We aim to enlarge scholarship about gaps between generational identity for LGBTQ + people since traditional generational theory has seldom been applied to LGBTQ+ people or communities.

In particular, we seek to extract from a considerably detailed investigation of the most recent six generations, a more nuanced understanding of how LGBTQ + members of various generational cohorts view the nation of segregated gay neighborhoods and how they have (or have not) contributed to sustaining gay neighborhoods to bestow them on subsequent generations of LGBTQ + people.

## Takeaway Messages

**Takeaway Message 1: Generational Worldview Shapes Gay Neighborhoods**

*Different generations of LGBTQ* + *individuals view and value gay neighborhoods differently.*

- Members of LGBTQ + generational cohorts can be identified according to a typical 20-year span. We argue that the process of achieving societal acceptance and winning civil rights may be different for each of the constituents under the LGBTQ + umbrella and that LGBTQ+ people experience “layered generations” based on their birth year and time when they came of age.
- Throughout their evolution, gay neighborhoods have been nurtured and sustained by LGBTQ + members of earlier generations (as shown in Fig. 14.8) for the generation that follows. Interest in gay neighborhoods, however, has begun to decrease among younger Millennials. We attribute this decline partly to different generational pressures—threats of terrorism, violence, and a general lack of a sense of collective safety—that have shaped lifestyle attitudes for this generation of young adults. In contrast to previous generations, many Millennials remain closer to home and retain close relationships with parents and family members. As LGBTQ + members of earlier generations encounter less social resistance to their LGBTQ+ identification or expression of sexual orientation, members of later generations may view gayborhoods as relics of the past or may find gay neighborhoods not to be welcoming in ways that match contemporary sensitivities toward inclusivity (Bitterman and Hess 2021).

**Takeaway Message 2: Gentrification May Be Killing Gay Neighborhoods**

*Gay neighborhoods are waning as older residents are selling or moving, and LGBTQ* + *people from younger generations are not replacing them.*

- This observation may be more related to real estate cost and the value placed on homeownership among members of the Millennial generation than about the value of LGBTQ-supportive community. As noted, generational differences in homeownership and living at home with parents longer is more common among Millennials than among previous generations (Bleemer et al. 2014).
- Gay neighborhoods were in their evolutionary infancy during the Baby Boom Generation and Generation X periods, and property was inexpensive during this early period. However, urban real estate demand has changed over time as gay neighborhoods have gentrified or hypergentrified ( Moss 2017). Often, LGBTQ + individuals that belong to earlier generations simply cannot afford to live in established gay neighborhoods, and living independently is often not a priority for those in earlier generations.
- Members of later generations also appear to be more comfortable discussing their gender identity and orientation with parents, family, and friends. The need to “run away” or physically re-locate to a gay neighborhood to find acceptance may be waning, but by staying behind in heteronormative neighborhoods, these young individuals may (perhaps unknowingly) be making these neighborhoods “ more gay.”

**Takeaway Message 3: More Recent Generational Cohorts Embrace Technology, and This Imperils Gay Neighborhoods**

*Technology allows later LGBTQ* + *generations to create* *virtual communities* *and has decreased the demand for and interest in* *gay neighborhoods*.

- Technology, perhaps more than any other factor, defines the generational divide. It has enabled a younger generation to socialize in a manner different from their elders. However, technology has also provided opportunities for members of older generations to stay connected. While technology is often cited as a potential reason for the possible decline of interest in gay neighborhoods among younger LGBTQ + individuals, this assessment is shortsighted because technology has also enabled many older LGBTQ+ individuals to remain connected despite advanced age. For example, LGBTQ + members of earlier generational cohorts may appreciate the nightlife that gay neighborhoods provide, but younger LGBTQ individuals also frequent gay bars, restaurants, and other gay neighborhood establishments technology in hand. For one generation, the attraction is place-driven, for another it may be place, driven by technology.
- Millennials and subsequent generations may place a different value on living among LGBTQ + community members in a gay neighborhood because technology lets them live anywhere and still actively communicate with the people with which they desire to associate. We note that various generations of LGBTQ+ individuals engage technological change differently, and the COVID-19 pandemic has further influenced the way nearly everyone engages technology (Miles 2021; Miles et al. 2021).

**Takeaway Message 4: Heteronormative Neighborhoods Become “More Gay” While Gay Neighborhoods Become “Less GayLess gay”**

*Millennials* *and later generations seem more comfortable disregarding societal expectations and constraints and less comfortable self*-*segregating into* *gay neighborhoods*.

- Over the years and as the generations progressed, some LGBTQ + individuals left gay neighborhoods, forced out in part by increasing housing costs related to gentrification and hypergentrification. To remain viable, many gay bars, restaurants, cafes, shops, and other gay-oriented establishments in gay neighborhoods adapted and welcomed people from more diverse groups (including straight people), making those neighborhoods “ less gay.” At the same time, mainstream bars, clubs, shops, and restaurants across the broader city began to more overtly welcome LGBTQ + individuals making those neighborhoods “ more gay.” As noted, Millennials typically experience less resistance than previous generations in expressing their sexual orientation and identity. They may be making heteronormative neighborhoods “ more gay” without being aware that they are doing so.

**Takeaway Message 5: Enhanced Civil Rights for Later Generations Stifle the Need for Gay Neighborhoods**

*Greater societal* *acceptance* *of LGBTQ* + *individuals makes more recent generations less likely to live in* *gay neighborhoods. There is consequently a view that many gay neighborhoods have lost their authenticity.*

**Fig. 14.9:**
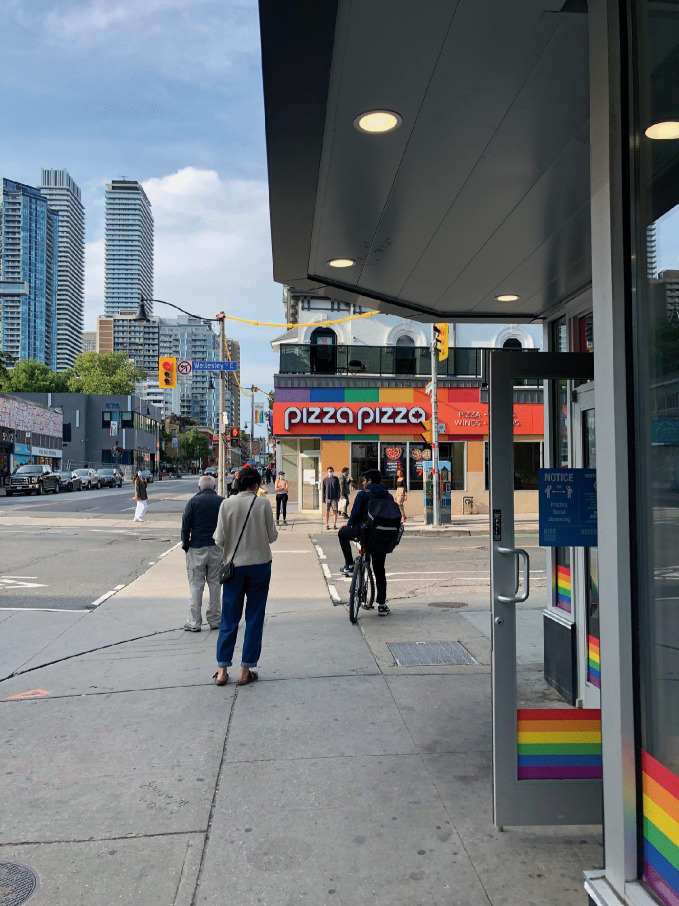
People of all ages—and from several generational cohorts—find common ground in the Church Street neighborhood in Toronto, Ontario, Canada (*Source* Image courtesy of Robert Modzelewski)
- Most gay neighborhoods were, for many years, centered around gay bars and nightlife that provided gathering space for sexual minorities. Gay neighborhoods have historically provided a degree of insulation from police brutality, hate-fueled violence, and harassment, especially among those misunderstood or ostracized by the mainstream. However, as LGBTQ + individuals enjoy greater civil rights and legal protections, social stigma related to identifying as LGBTQ+ has decreased. Compared to generations past, younger LGBTQ + individuals tend to enjoy a greater degree of familial support when identifying as a sexual minority . Cultural shame associated with LGBTQ + status in the Silent and Greatest Generations has diminished and has been replaced for subsequent generations by pride. These changes are markedly generational.
- Significantly, with the legal right of same-sex couples to marry, the gay family model has transformed. For example, a gay baby boomer likely has a somewhat different nuclear family make-up than a millennial gay man may have or may wish to have. A more traditional family structure (two married adults with children) is becoming more common in LGBTQ + communities, and this may serve to change the flavor of gay neighborhoods as LGBTQ+ families seek amenities (such as daycare, schools, and family-centered medical care) that were not traditionally associated with gayborhoods. However, this shift does not mean that gay neighborhoods are dead or dying. LGBTQ + individuals recognize gay neighborhoods as the center of gay culture and will often socialize and celebrate in these locations. Meanwhile, the need to seek refuge in an urban gay neighborhood has diminished because LGBTQ + individuals continue to proudly fight for equality and civil rights, ensuring that smaller cities and towns are more inclusive of LGBTQ + people as residential settlements everywhere become “ more gay” through a diffusion of formerly concentrated LGBTQ + communities.

- Younger LGBTQ+ individuals from more recent generational cohorts have come of age in a time when being gay is broadly accepted throughout mainstream culture, and LGBTQ+ individuals enjoy greater recognition and enhanced civil rights and legal protections. As a result, the desire to purposely isolate with like people for protection in specific geographiclocations has seemingly diminished. Millennials—whether LGBTQ+ or not—are likely to behave more uniformly regarding housing preferences and choices about neighborhoods and cities for their residential location (Nash 2013). These observations suggest broader societal shifts, not necessarily a diminished demand for or interest in gay neighborhoods (Fig. 14.9).
